# Inhibitory Activities of Rare Ginsenoside Rg4 on Cecal Ligation and Puncture-Induced Sepsis

**DOI:** 10.3390/ijms231810836

**Published:** 2022-09-16

**Authors:** Go Oun Kim, Nayeon Kim, Gyu Yong Song, Jong-Sup Bae

**Affiliations:** 1 Research Institute of Pharmaceutical Sciences, College of Pharmacy, Kyungpook National University, 80 Daehak-ro, Buk-gu, Daegu 41566, Korea; 2 College of Pharmacy, Chungnam National University, 99 Daehak-ro, Yuseong-gu, Daejon 34134, Korea

**Keywords:** ginsenoside Rg4, CLP, sepsis, kidney injury, inflammation

## Abstract

Sepsis is an uncontrolled response to inflammatory infection and is associated with high levels of mortality and morbidity. Rg4 is a rare ginsenoside mainly found in the leaves of *Panax ginseng* C. A. Meyer and the major protopanaxatriol-type ginsenoside of black ginseng. In this study, we determined whether Rg4 affects cecal ligation and puncture (CLP)-induced sepsis. Animals were separated into the following six groups: control group, CLP-operated group, CLP plus maslinic acid (MA), and CLP plus Rg4 (5, 10, or 15 mg/kg). Survival rate, body weight changes, inflammatory cytokines, and histological analyses were assessed. Human endothelial cells were activated with the high-mobility group box 1 (HMGB1) protein and Rg4. Cell viability was determined using the 3-(4,5-dimethylthiazol-2-yl)-2,5-diphenyltetrazolium bromide (MTT) assay. Enzyme-linked immunosorbent assay (ELISA) and Western blot analysis were used to assess inflammation and gene expression, respectively. After CLP surgery, the Rg4-administered group exhibited a higher survival rate and body weight compared with the untreated control group. Rg4 treatment reduced cytokine levels, including tumor necrosis factor (TNF)-α and interleukin (IL)-1β, as well as nitric oxide (NO) levels and renal inflammation. After Rg4 treatment of HMGB1-activated cells, the expressions of toll-like receptor (TLR) 4 and TNF-α were decreased, and the activation of phosphoinositide 3-kinase (PI3K)/AKT signaling increased cell viability. In summary, Rg4 inhibited inflammation and exhibited a protective effect against CLP-induced sepsis, thereby reinforcing cell survival against septic responses.

## 1. Introduction

Ginseng, the root of *Panax ginseng* Meyer, has long been used as a dietary supplement and traditional medicine in East Asian countries. Ginseng is effective at enhancing vitality, boosting immunity, preventing cancer, strengthening body resistance, and treating diabetes and fatigue [1,2,3,4]. As the main component of ginseng, ginsenosides have been isolated from red and white ginseng and structurally confirmed. Black ginseng is prepared by steaming and drying ginseng nine times. It has recently attracted attention from scientists for its diverse pharmacological effects, including wound healing, antidiabetic, immune enhancement, and antioxidant activities [5,6,7]. During the process of preparing black ginseng, the structure of the ginsenosides is altered to a low molecular weight consisting of low-polarity rare ginsenosides that undergo hydrolysis, isomerization, and dehydration at C-20. Hydrolysis also occurs at C-3 or C-6 of the aglycone skeleton [8]. Rg4, a rare ginsenoside, is the main low-polarity protopanaxatriol-type ginsenoside of black ginseng. Recently, the rare ginsenoside Rg4 was shown to exhibit potent antistress, antisubstrate metalloproteinase-13 expression, pulmonary protection, and apoptosis-inducing effects [9,10,11,12].

Sepsis is the result of an uncontrolled host response to infection and causes life-threatening organ dysfunction. It is the leading cause of death from infection, especially if not recognized and treated immediately [13,14]. Sepsis is characterized by whole-body inflammation known as “systemic inflammatory response syndrome” or SIRS. It is increasingly responsible for common illnesses and deaths, especially in older adults, immunocompromised, and severely ill patients, and hospitalization for sepsis has more than doubled in the past decade, and more than 50% of hospital deaths are associated with sepsis [13,14,15]. Thus, sepsis has become a significant health and economic burden worldwide. Furthermore, pattern recognition receptors (PRRs) are typical innate immune sensors that recognize a variety of pathogen-associated molecular patterns (PAMPs) and damage-associated molecular patterns (DAMPs) [13,16,17]. Overproduction of PAMP and DAMP sensors during sepsis is implicated in multiple organ failure (MOF) [13,16,17]. Xigris, used to treat sepsis, was withdrawn from the market in 2011 due to issues with side effects and lack of efficacy [18]. Since then, there has been no approved treatment for sepsis. Therefore, the development of alternative drugs for sepsis has becomes imperative.

Cecal ligation and puncture (CLP) surgery is the most common technique for experimentally inducing sepsis in rodents and is currently considered the standard method for sepsis studies [19,20]. Although the CLP model was developed more than 40 years ago, it is a realistic model for inducing polymicrobial sepsis for the study of sepsis mechanisms and treatment [19,20]. Since no studies exist with respect to the relationship between Rg4 and sepsis, we determined the effect of Rg4 on CLP-induced sepsis. We also propose that the Rg4 pathway may be used for protection against CLP-induced septic mice.

## 2. Results

### 2.1. Protective Effects of Rg4 on CLP-Induced Sepsis

**Figure 1:**
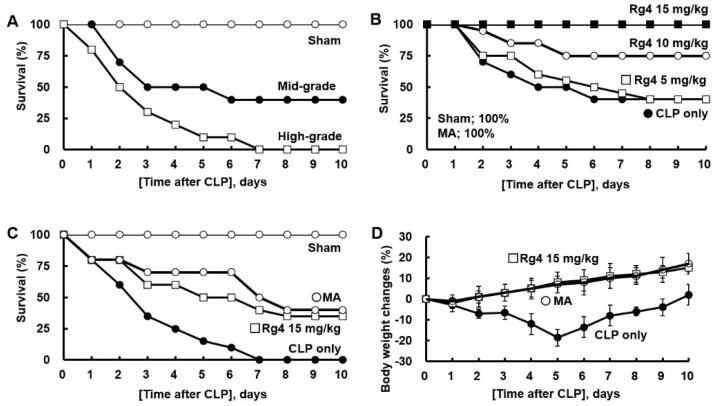
Protection of mice from the morbidity and mortality of sepsis by Rg4 after the CLP procedure. (**A**) Survival rates after the induction of different (mid or high) severity grades of sepsis by CLP. (**B**,**C**) Each group consisted of 10 mice and received intravenous injections of vehicle, MA (0.7 mg/kg) or Rg4 (5, 10, or 15 mg/kg) in 0.5% DMSO at 24 h after mid- (**B**) or high-grade (**C**) CLP. Survival rates of challenged mice were monitored for 10 days. (**D**) Body weight changes were measured during the period in Figure (**B**). All values are presented as the mean ± SD of three separate experiments. * *p* < 0.01 vs. CLP group for Rg4 or MA in figure (**D**).

The severity of sepsis resulting from CLP surgery depends on which area of the cecum is punctured, which leads to mid- or high-grade mortality [21]. Figure 1A shows the survival rates of mid- or high-grade CLP in mice. The data indicate that in high-grade sepsis, all mice died, whereas there was a 40% survival rate in midgrade sepsis, which is consistent with that of a previous study [21]. The mid- and high-grade CLP surgery was used to determine the effect of Rg4 on CLP-induced sepsis. Rg4 (5, 10, or 15 mg/kg) or 0.5% DMSO was injected into mice as a negative control, or MA (0.7 mg/kg) was injected as a positive control [22]. When Rg4 was administered at 10 or 15 mg/kg to under-mid-grade sepsis, 75% or 100% of the mice survived for 10 days, respectively (Figure 1B). However, the administration of 5 mg/kg of Rg4 did not affect survival compared with the control group (Figure 1B). Furthermore, the positive control group, which was injected with MA, exhibited a 100% survival rate (Figure 1B). These results suggest that Rg4 administration at a concentration of 15 mg/kg protects mice from CLP-induced sepsis. Therefore, we used 15 mg/kg of Rg4 in subsequent antiseptic experiments. Next, Rg4 (15 mg/kg) was evaluated in a high-grade sepsis model (resulted in 100% death, Figure 1A). The survival rate was significantly improved (*p* < 0.01) compared with the control group (Figure 1C). This indicates that Rg4 (15 mg/kg) can protect mice from CLP-induced sepsis even in a high-grade sepsis model. The administration of Rg4 (15 mg/kg) also resulted in effects on weight loss (down to −18.5%) in a midgrade CLP model. Body weight was restored by up to 15% following Rg4 treatment at 15 mg/kg.

### 2.2. Inhibitory Effects of Rg4 on Inflammatory Cytokine Levels and Renal Inflammation in CLP-Operated Mice

**Figure 2:**
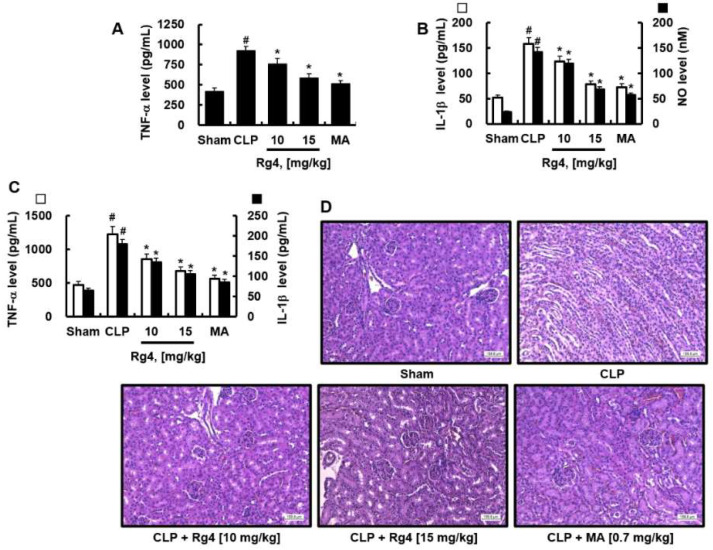
Inhibition of inflammatory cytokines and renal and hepatic inflammation by Rg4 in CLP. Each group consisted of 5 mice, which received intravenous injections of vehicle, MA (0.7 mg/kg) or Rg4 (5, 10, or 15 mg/kg) in 0.5% DMSO at 24 h after high-grade CLP. At 72 h after CLP, the kidneys or livers were homogenized and used for the determination of TNF-α (**A**) or IL-1β (**B**) in kidneys (**A**,**B**) or livers (**C**). All values are presented as the mean ± SD of three separate experiments. # *p* < 0.01 vs. control group or * *p* < 0.01 vs. CLP group. (**D**) Kidney tissues were stained with H&E. Photomicrographs of kidney tissues at 72 h after CLP are shown (original magnification, ×200); the images are representative of three independent experiments. Scale bars: 160 μm.

We determined whether Rg4 affects inflammatory cytokine production in CLP-operated mice. The administration of Rg4 reduced cytokine levels, including TNF-α, IL-1β, and NO in the kidneys, the most sensitive organ to sepsis [23] resulting from high-grade CLP (Figure 2A,B). Rg4 also reduced the levels of TNF-α and IL-1β in the liver, which supported the anti-inflammatory activities of Rg4 on sepsis. These results indicate that Rg4 acts as an anti-inflammatory agent in vivo. Next, we confirmed the effects of Rg4 on renal inflammation following H&E staining of the kidneys. The results indicated that Rg4 treatment reduced inflammation compared with the untreated group (Figure 2D). This indicates that Rg4 attenuates CLP-induced renal inflammation by decreasing the production of inflammatory cytokines.

### 2.3. Inhibitory Effects of Rg4 on TLR and NF-κB Expression

**Figure 3:**
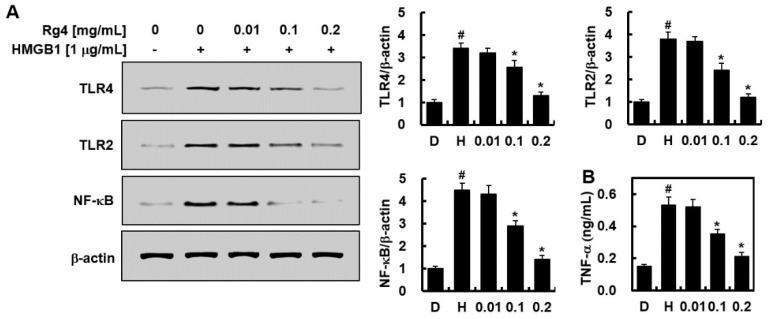
Inhibition of HMGB1-induced TLR/NF-κB expression by Rg4 in vitro. (**A**) HUVECs were activated with HMGB1 (1 μg/mL) treatment for 6 h, followed by treatment with various concentrations of Rg4 for another 6 h. Cell lysates were subjected to Western blot analysis to determine the protein levels of TLR2, TLR4, and NF-κB. Representative Western blots (left) and relative densitometric quantification (right) of each gene. (**B**) Supernatants from (**A**) were used for TNF-α ELISA. All values are presented as the mean ± SD of three separate experiments. D = 0.5%, DMSO was the vehicle control and H = HMGB1. # *p* < 0.01 vs. DMSO or * *p* < 0.01 vs. HMGB1 group.

HUVECs were activated with HMGB1 as a late sepsis mediator [13,24], followed by treatment with Rg4 for 6 h [12]. TLR expression was measured by Western blot analysis. Rg4 (0.1 or 0.2 mg/mL) treatment significantly reduced both TLR2 and TLR4 expression compared with the untreated control group (Figure 3A). Treatment with Rg4 (0.1 or 0.2 mg/mL) also suppressed NF-κB expression, the canonical downstream gene of the TLR pathway (Figure 3A). The average circulating blood volume for mice is 72 mL/kg [25]. Because the average weight of a used mouse is 27 g, and the average blood volume is 2 mL, the amount of Rg4 (0.1 or 0.2 mg/mL) injected yielded a maximum concentration of 7.5 or 15 mg/kg in the peripheral blood. In addition, the production of TNF-α was markedly reduced when HUVECs were treated with Rg4 (Figure 3B), indicating that Rg4 suppressed the activation of TLR after HMGB1 activation. Thus, Rg4 inhibits cytokine production and NF-κB expression.

### 2.4. Effects of Rg4 on Cell Survival In Vitro

**Figure 4:**
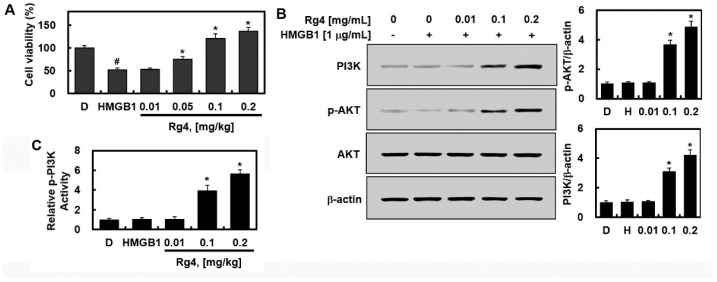
Increased cell survival through induction of PI3K/AKT signaling by Rg4 in HMGB1-mediated sepsis in vitro. HUVECs were activated with HMGB1 (1 μg/mL) for 6 h and then treated with various concentrations of Rg4 for another 6 h. (**A**) The MTT assay was performed to assess cell viability. Cell lysates were subjected to Western blot analysis to estimate protein levels of PI3K, p-AKT, and total AKT (**B**) or to ELISA for phosphor-PI3K (**C**). Representative Western blots (left) and relative densitometric quantification (right) of each gene. All values are presented as the mean ± SD of three separate experiments. D = 0.5%, DMSO was the vehicle control and H = HMGB1. # *p* < 0.01 vs. DMSO or * *p* < 0.01 vs. HMGB1 group.

Previous studies have shown that Asian or Korean ginseng itself or several types of ginsenosides isolated from Asian or Korean ginseng increase cell survival [26,27,28] by activating the PI3K/AKT pathway under conditions of oxidative stress [29]. Therefore, we determined whether Rg4 increases the survival of HUVECs during HMGB1-induced septic conditions. To confirm this, we conducted cell viability assays following HMGB1 activation with and without Rg4 treatment. HMGB1 reduced the viability of HUVECs, whereas Rg4 (0.1 or 0.2 mg/mL) treatment increased cell viability (Figure 4A). This indicates that treatment with Rg4 increases cell viability under HMGB1-induced septic conditions. With respect to PI3K/AKT signaling, PI3K levels were unchanged following HMGB1 activation, but were markedly increased after Rg4 treatment (Figure 4B). Furthermore, after Rg4 treatment, significantly upregulated p-AKT expression (Figure 4B) or p-PI3K (C) was observed compared with that of the nonactivated control group. However, total AKT levels were unchanged (Figure 4B), suggesting that the improved cell viability induced by Rg4 is enhanced by PI3K/AKT activation.

## 3. Discussion

Sepsis is a fairly heterogeneous syndrome caused by an uncontrolled immune response to infection and leads to organ failure [13,14]. There are many causes of sepsis, including bacterial, viral, and fungal infections, and it is the leading cause of death in ICU patients [13,14]. TNF-α, IL-1β, and IL-6 are the major inflammatory factors that could contribute to sepsis [13,14]. Xigris is the only FDA-approved drug for the treatment of sepsis; however, in 2011, Eli Lilly (Indianapolis, IN, USA) withdrew it from the global market because of negative results obtained in a septic shock test in 1700 patients [30]. There is currently a shortage of drugs approved for the treatment of sepsis following the withdrawal of Xigris. Therefore, it is necessary to develop a new and safe treatment method for sepsis. In the present study, Rg4 suppressed NO production and downregulated inflammatory cytokine levels, including TNF-α and IL-1β in CLP-induced septic mice.

To identify the potential mechanisms of Rg4 in regulating the production of inflammatory cytokines, we measured the expression of TLR-2,4 and NF-κB in HUVECs. TLRs play an important role in regulating inflammatory cytokine production during the septic response. Furthermore, the expression of TLR-2,4 at early stages of sepsis correlates with mortality [31]. NF-κB is an important factor in TLR signaling as it regulates the pathogenesis of pneumococcal sepsis. Our data indicate that septic conditions induced by HMGB1 increased the expression of TLR-2,4 and NF-κB, whereas Rg4 treatment reduced the response. Rg4 treatment ameliorated renal inflammation in CLP-induced sepsis and exhibited a higher survival rate compared with the untreated control group. Thus, Rg4 ameliorated TLR-mediated inflammation and subsequent sepsis induced by CLP surgery. The results clearly demonstrate the protective effects of Rg4 against CLP-induced sepsis.

The PI3K/AKT signaling pathway plays an important role in cell survival [32]. In the response to sepsis, HMGB1 activation induced cell death, whereas Rg4 treatment enhanced cell survival by increasing PI3K/p-AKT expression. Thus, the upregulation of PI3K/AKT signaling may enhance cellular resistance to severe inflammatory responses induced by HMGB1. Moreover, the inhibition of the PI3K/AKT pathway results in the increased production of cytokines and chemokines, indicating that the stimulation of the PI3K/AKT pathway may be beneficial for the treatment of sepsis. Thus, the activation of the PI3K/AKT signaling pathway by Rg4 during sepsis may inhibit inflammation and increase cell survival. Rg4 may play a dual role by inhibiting inflammatory cytokine production and enhancing cell survival.

The Rg4-treated group exhibited a higher survival rate and lower morbidity compared with the untreated control group. In conclusion, Rg4 improves cell survival and reduces the production of inflammatory cytokines, thereby attenuating the lethality and mortality associated with sepsis. Rg4 may provide a therapeutic benefit for severe inflammatory diseases associated with sepsis by enhancing immunity.

## 4. Materials and Methods

### 4.1. Cell Culture and Reagents

Primary human umbilical vein endothelial cells (HUVECs) were purchased from Cambrex Bio Science (Charles City, IA, USA) and maintained as described previously [33,34]. MTT, dimethyl sulfoxide (DMSO), and MA were obtained from Sigma Chemical Co. (St. Louis, MO, USA). MA was used as a positive control [22]. Human recombinant HMGB1 and rare ginsenoside Rg4 (M.W. of Rg4 is 767) were obtained from Abnova (Taipei City, Taiwan) and Arez Co., Ltd. (Sejong, Korea), respectively. Rg4 purity was determined to be greater than 98% by HPLC [12].

### 4.2. Animals and the CLP Procedure

Male C57BL/6 mice (8–10 weeks old, average weight of 27 g) were purchased from the Orient Bio Co. (Seongnam, Korea). The mice were housed according to standard animal care conditions and allowed to eat and drink freely. All care was performed in accordance with the Guide for the Care and Use of Laboratory Animals. CLP surgery on the animals (mid- or high-grade) was performed as previously described [21,22,33]. To induce sepsis, a 2 cm midline incision was made to allow exposure of the cecum to the adjoining intestine. The cecum was then tightly ligated with a 3.0 silk suture 5.0 mm from the cecal tip and punctured once for midgrade or twice for high-grade with a 22-gauge needle. The cecum was then gently squeezed to extrude a small amount of feces from perforation sites and returned to the peritoneal cavity. The laparotomy site was then closed using a 4.0 silk suture. In sham control animals, the cecum was exposed but not ligated or punctured, and then returned to the abdominal cavity. The cecum of the sham control group was exposed, but not ligated or punctured. This protocol was approved by the Animal Care Committee at Kyungpook National University prior to conducting the study (IRB No. KNU 2019-103).

### 4.3. Experimental Design

The mice were divided into six groups (*n* = 20) as follows: control sham group, CLP, CLP plus MA (0.7 mg/kg), and CLP plus Rg4 (5, 10, or 15 mg/kg). Drugs or saline was intravenously injected 24 h after CLP surgery. Blood was collected from the orbital venous plexus 24 h after the administration of drugs or saline, then centrifuged at 3000× *g* at 4 °C for 10 min. Serum was stored at −80 °C.

### 4.4. Hematoxylin and Eosin (H&E) Staining and Histopathological Examination

Twenty-four hours after drug administration, kidney samples were fixed in 10% (*v*/*v*) phosphate-buffered formalin for 24 h and embedded in paraffin. Sections (4 μm) were cut using a microtome, stained with hematoxylin and eosin, and observed using a light microscope as previously described [12,34,35].

### 4.5. Biochemical Measurement

TNF-α and IL-1β concentrations in kidney or liver tissues were measured using ELISA kits (R&D Systems, Minneapolis, MN, USA) as previously described [36]. The renal NO concentration was determined by the nitric acid reductase method [37,38,39]. Phosphorylated PI3K levels were measured using ELISA kits (MyBioSource, San Diego, CA, USA).

### 4.6. Cell Viability Assay

HMGB1 was used to activate HUVECs, and after 6 h of activation, the cells were treated with Rg4 for 48 h, followed by the MTT assay to estimate cell viability [35,39].

### 4.7. Western Blot Analysis

Cytoplasmic extraction reagents (Thermo Fisher Scientific, Waltham, MA, USA) were used for cytoplasmic protein isolation according to the manufacturer’s instructions. Protein (20 µg) was added to each well and separated by 10% sodium dodecyl sulfate–polyacrylamide gel electrophoresis. The proteins were electroblotted onto Immobilon^®^ membranes (Millipore, Billerica, MA, USA) overnight. The membranes were blocked in 5% low-fat milk powder in Tris-buffered saline (50 mM Tris-HCl and 150 mM NaCl; pH 7.5) containing 0.05% Tween 20 for 1 h. After blocking, the membranes were incubated with toll-like receptor (TLR)2, TLR4, PI3K, p-AKT, nuclear factor (NF)-κB, or total AKT (Santa Cruz Biotechnology, Santa Cruz, CA, USA). They were then incubated with horseradish-peroxidase-conjugated secondary antibody, followed by enhanced chemiluminescence detection according to the manufacturer’s instructions. Protein concentrations were determined using the ImageJ gel analysis tool (NIH, Bethesda, MD, USA) and expressed in units of measure of relative density of the corresponding β-actin control.

### 4.8. Statistical Analysis

For each assay, more than three independent experiments were performed. Statistical analyses were performed using ANOVA, followed by Student’s *t*-test. All values are expressed as the mean ± standard deviation (SD). Statistical significance was accepted at *p*-values < 0.05.
